# Suppression of Lipid Accumulation in the Differentiation of 3T3-L1 Preadipocytes and Human Adipose Stem Cells into Adipocytes by TAK-715, a Specific Inhibitor of p38 MAPK

**DOI:** 10.3390/life13020412

**Published:** 2023-02-01

**Authors:** Nivethasri Lakshmana Perumal, Amila Mufida, Anil Kumar Yadav, Dae-Gu Son, Young-Wook Ryoo, Sung-Ae Kim, Byeong-Churl Jang

**Affiliations:** 1 Department of Molecular Medicine, College of Medicine, Keimyung University, Daegu 42601, Republic of Korea; 2 The Hormel Institute, University of Minnesota, Austin, MN 55912, USA; 3 Department of Plastic Surgery, College of Medicine, Keimyung University, Daegu 42601, Republic of Korea; 4 Department of Dermatology, College of Medicine, Keimyung University, Daegu 42601, Republic of Korea

**Keywords:** TAK-715, p38 MAPK, PPAR-γ, adipogenesis, 3T3-L1, hASCs

## Abstract

**Simple Summary:**

p38 MAPK is emerging as a novel regulator in (pre)adipocyte differentiation. TAK-715 is a selective inhibitor of p38 MAPK, but its anti-adipogenic effect and mechanism during the differentiation of preadipocytes and human adipose stem cells (hASCs) into adipocytes remain unclear. In this study, a treatment with TAK-715 (10 μM) led to a marked inhibition of lipid accumulation during the adipocyte differentiation of 3T3-L1 preadipocytes and hASCs. TAK-715 further downregulated the expression and phosphorylation of C/EBP-α, PPAR-γ, STAT-3, FAS, perilipin A, p38 MAPK, and its downstream effector ATF-2 during 3T3-L1 cells and hASCs differentiation. Taken together, this is the first report that shows TAK-715 has a strong lipid-lowering effect during the adipocyte differentiation of 3T3-L1 preadipocytes and hASCs by regulating the expression and phosphorylation of C/EBP-α, PPAR-γ, STAT-3, FAS, perilipin A, ATF-2, and p38 MAPK. In this work, TAK-715 is proposed as a potential preventive or therapeutic agent for managing obesity.

**Abstract:**

Excessive preadipocyte differentiation is linked with obesity. Although previous studies have shown that p38 MAPK is associated with adipogenesis, the regulation of preadipocyte differentiation by TAK-715, an inhibitor of p38 mitogen-activated protein kinase (MAPK), remains unclear. Interestingly, TAK-715 at 10 μM vastly suppressed the accumulation of lipid and intracellular triglyceride (TG) content with no cytotoxicity during 3T3-L1 preadipocyte differentiation. On mechanistic levels, TAK-715 significantly decreased the expressions of the CCAAT/enhancer-binding protein-α (C/EBP-α), peroxisome proliferator-activated receptor gamma (PPAR-γ), fatty acid synthase (FAS), and perilipin A. Similarly, the phosphorylation of the signal transducer and activator of transcription-3 (STAT-3) in differentiating 3T3-L1 cells was also reduced with TAK-715 treatment. Moreover, TAK-715 significantly blocked the phosphorylation of activating transcription factor-2 (ATF-2), a p38 MAPK downstream molecule, during 3T3-L1 preadipocyte differentiation. Of importance, TAK-715 also markedly impeded the phosphorylation of p38 MAPK and suppressed lipid accumulation during the adipocyte differentiation of human adipose stem cells (hASCs). Concisely, this is the first report that TAK-715 (10 μM) has potent anti-adipogenic effects on the adipogenesis process of 3T3-L1 cells and hASCs through the regulation of the expression and phosphorylation of p38 MAPK, C/EBP-α, PPAR-γ, STAT-3, FAS, and perilipin A.

## 1. Introduction

Obesity is linked with changes in the adipose tissue that lead to metabolic dysfunction [1]. Obesity gives rise to several pathological conditions, such as dyslipidemia, type 2 diabetes mellitus (T2DM), and cardiovascular diseases [2]. The differentiation of preadipocytes, also known as adipogenesis, is a multi-complex way of storing excess energy in the structure of triglyceride (TG) and playing a part in expanding the adipose tissue mass of obesity [3]. Hence, any compound that can repress enormous adipogenesis may be considered a promising preventive and/or therapeutic strategy to overcome obesity and associated diseases.

Adipogenesis is tightly modulated by the transcriptional cascade and signaling pathways [4]. At an early stage of preadipocyte differentiation, the expression of CCAAT/enhancer-binding proteins (C/EBP), including C/EBP-δ and C/EBPβ, are elevated temporarily [2]. Throughout the middle phase of cell differentiation, C/EBP-β/δ further induces the main transcription factors of adipogenesis, such as C/EBP-α and peroxisome proliferator-activated receptor-γ (PPAR-γ) [4]. PPAR-γ and C/EBP-α synergistic work then leads to the differentiation and causes the promotion of adipogenic genes, including lipoprotein lipase (LPL), acetyl CoA carboxylase (ACC), adipocyte protein 2 (aP2), fatty acid synthase (FAS), and perilipin in the terminal stage of cell differentiation [2,5,6,7]. In addition, early reports point out that the increased phosphorylation and expression of the signal transducer and activator of transcription-3/5 (STAT-3/5) family takes part in the transcriptional control at the time of the differentiation process of 3T3-L1 preadipocyte [8]. Activating transcription factor-2 (ATF-2), a member of the ATF family of transcription factors, is expressed in various tissues, including white adipose tissue. Abundant studies also indicate that ATF-2 is stimulated by p38 mitogen-activated protein kinase (MAPK) to control preadipocyte differentiation and fat storage [9,10,11].

TAK-715 (N-[4-[2-Ethyl-4-(3-methylphenyl)-1, 3-thiazol-5-yl[-2-pyridyl[benzamide) is an inhibitor of p38 MAPK [12,13]. At present, the TAK-715 regulation of preadipocyte differentiation is unknown. In this study, we explored the effects of TAK-715 on lipid accumulation in 3T3-L1 preadipocyte and human adipose stem cells (hASCs) adipocyte differentiation. For the first time, we demonstrate that TAK-715 at 10 μM strongly suppresses lipid accumulation in the time of 3T3-L1 cells and hASCs differentiation, which are interfered with through the reduction of expression and phosphorylation levels of C/EBP-α, PPAR-γ, STAT-3, FAS, perilipin A, ATF-2, and p38 MAPK.

## 2. Materials and Methods

### 2.1. Materials

TAK-715 was purchased from Selleckchem (Houston, TX, USA). TAK-715 was dissolved in 100% DMSO and prepared as a 10 mM stock solution. Dexamethasone, 3-Isobutyl-1-methylxanthine (IBMX), insulin, and Oil red O stock solution were obtained from Sigma (St. Louis, MO, USA). Enhanced chemiluminescence (ECL) reagents were purchased from Advansta (San Jose, CA, USA). A detailed list of antibodies used in this study is included in Table 1.

### 2.2. 3T3-L1 Cells or hASCs Culture and Differentiation 

Murine 3T3-L1 cells (ATCC, Manassas, VA, USA) were cultured in a growth media containing Dulbecco’s Modified Eagles’ Medium (DMEM), 10% heat-inactivated fetal calf serum (Gibco, Grand Island, NY, USA), and 1% penicillin–streptomycin (Welgene, Daegu, Republic of Korea). The adipocyte differentiation was induced by changing the medium to DMEM supplemented with 10% fetal bovine serum (FBS) (Welgene, Daegu, Republic of Korea) added with a cocktail of hormones (MDI) that include 0.5 mM IBMX (M), 0.5 µM dexamethasone (D), and 5 µg/mL insulin (I) either with or without TAK-715 at the indicated concentrations. On day 2, the first differentiation medium was replaced with DMEM supplemented with 10% FBS and 5 µg/mL insulin either with or without TAK-715 at the indicated doses for an additional 3 days. The cells were further fed with DMEM containing 10% FBS in the presence or absence of TAK-715 for an additional 3 days. On day 8, the preadipocytes became mature adipocytes that were rounded up and filled with lipid droplets. 

The hASCs were isolated from abdominal subcutaneous adipose tissue of female patients admitted to Keimyung University Dongsan Hospital (KUDH), Daegu, Republic of Korea. The Ethics Committee of KUDH approved the study protocol (No. 2021-02-063-018), and the informed consent was obtained from the patients. The hASCs isolated were then cultured in growth media containing Dulbecco’s Modified Eagles’ Medium/ Nutrient Mixture F-12 (DMEM/F-12) supplemented with 10% heat-inactivated fetal bovine serum, 1% penicillin-streptomycin, and 0.25 μg/mL fungizone. The adipocyte differentiation of hASCs was induced by using adipocyte differentiation medium (Zenbio, DM-2) for 7 days, and then the medium was changed into adipocyte maintenance medium (Zenbio, AM-1) until 5 days in the absence or presence of TAK-715 at the indicated concentrations. On day 12, hASCs became mature adipocytes that rounded with large lipid droplets in the cytoplasm.

### 2.3. Oil Red O Staining 

Lipids detection in mature adipocytes of 3T3-L1 cells and hASCs were stained with the Oil Red O staining method. Cells were washed twice with phosphate-buffered saline (PBS) and fixed with 10% formaldehyde for 2 h. Furthermore, cells were washed with 60% isopropanol and dried. The fixed cells were then stained with Oil Red O working solution (Sigma, St. Louis, MO, USA) for 1 h at room temperature (RT) and washed twice with distilled water. Stained LDs were visualized under light microscopy (Nikon, TS100, Tokyo, Japan). 

### 2.4. Cell Count Analysis

The 3T3-L1 preadipocytes or hASCs were seeded in 24-well plates. Under the differentiation conditions mentioned above, cells grew similarly. Trypan blue was used to visualize the differentiation of the control or TAK-715-treated 3T3-L1 cells or hASCs on days 8 or 12, respectively. Only cells with intact membranes can effectively block the dye; after that, dying cells with damaged membranes become stained and must be counted under a light microscope. The cell count assay was performed three times. The data represent the average and standard error (SE) of three independent studies.

### 2.5. Measurement of Intracellular TG Content

Under the differentiation with the above conditions, cells grew similarly. A commercially available AdipoRed test reagent kit was used to assess the intracellular TG level in the control or TAK-715-treated 3T3-L1 cells on day 8 of differentiation under the manufacturer’s instructions (Lonza, Basel, Switzerland). On Victor3 (Perkin Elmer, Waltham, MA, USA), fluorescence was measured with an excitation at 485 nm and an emission at 572 nm. The results are the mean SE of three independent experiments.

### 2.6. Preparation of Whole-Cell Lysates 

At the predetermined time, 3T3-L1 cells or hASCs were washed twice with PBS before being incubating in a RIPA buffer containing a proteinase inhibitor cocktail (1x). In a 1.5 mL tube, the cell lysates were collected and centrifuged at 12,000 rpm for 20 min at 4 °C. Protein concentrations were calculated using the Bradford reagent after the supernatant was preserved (Bio-Rad, Hercules, CA, USA). 

### 2.7. Westerm Blotting Analysis 

Proteins were separated with 10% SDS-PAGE and transferred to nitrocellulose membranes (MilliporeSigma, Burlington, MA, USA). The membranes were washed with TBS (10 mM Tris, 150 mM NaCl) supplemented with 0.05% (vol/vol) Tween-20 (TBST) and further blocked with TBST containing 5% skimmed milk powder. The membranes were incubated overnight with specific primary antibodies listed in Table S1 at 4 °C. The membranes were then incubated with secondary antibodies coupled to horseradish peroxidase at RT for 2 h. All membranes were washed three times with TBST at RT. Eventually, the membranes were developed using ECL reagents. The expression level of actin was used to determine equal protein loading.

### 2.8. RT-qPCR

Total RNA from the control or TAK-715-treated 3T3-L1 cells was extracted using RNAiso Plus (TaKaRa, Kusatsu, Shiga, Japan). Subsequently, random hexadeoxynucleotide primer and reverse transcriptase were used to reverse the transcription of the RNA into complementary DNA (cDNA). Quantitative gene transcript levels were determined using SYBR green (TaKaRa, Kusatsu, Shiga, Japan) with LightCycler96 Machine (Roche, Mannheim, Germany). PCR reactions were performed three times for each sample, and transcript levels of each gene were normalized to the expression level of 18S rRNA. Primer sequences used in this study are listed in Table 2. Results are presented as the mean ± SE of three independent studies.

### 2.9. Statistical Analysis

Cell count analysis was carried out in triplicate and repeated three times. The results were presented as the mean ± standard error (SE). One-way ANOVA was used to evaluate the significant differences between all groups tested. All statistical analyses were elucidated on a value of *p* < 0.05.

## 3. Results

### 3.1. TAK-715 at 10 μM Markedly Suppresses Intracelullar Lipid Accumulation and TG Content during the Adipogenesis of 3T3-L1 Preadipocyte

**Figure 1:**
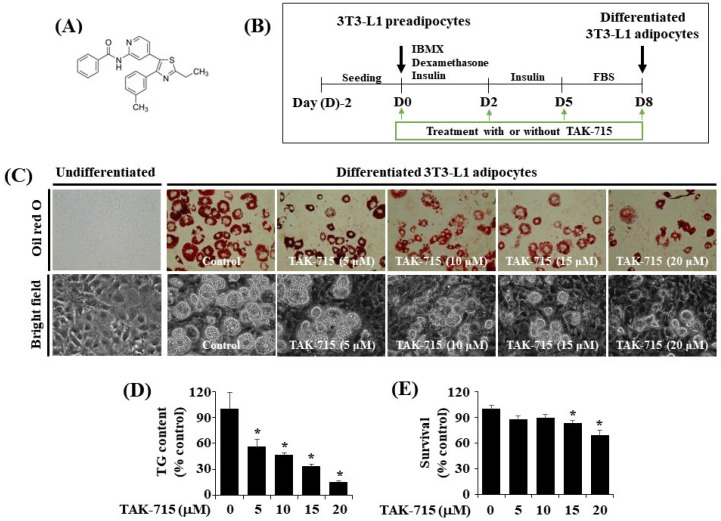
TAK-715 effect on lipid droplet accumulation, triglyceride (TG) levels, and cell growth (survival) during the adipogenesis of 3T3-L1 cells. (**A**) The chemical structure of TAK-715. (**B**) The experimental design for 3T3-L1 cells differentiation. (**C**) Representative images of intracellular lipid droplet (LD) formation in 3T3-L1 preadipocyte (D0) and adipocyte (D8) that were grown in the absence or presence of TAK-715 at various concentrations by Oil Red O staining (upper panels) and phase-contrast image (lower panels) at 400× magnification. (**D**) Intracellular TG content in 3T3-L1 adipocyte (D8) that were grown with or without of TAK-715 at all tested concentrations using AdipoRed assay. All experiments were performed independently in triplicate. The bars indicate mean ± standard error (SE) of three independent experiments. * *p* < 0.05 compared to the s control samples. (**E**) Cell count assay was used to determine the total number of living cells in control or differentiated 3T3-L1 cells on D8 treated with TAK-715. The bars indicate mean ± SE from three independent experiments, each done in triplicate. * *p* < 0.05 vs. undifferentiated 3T3-L1 cells.

The chemical structure of TAK-715 is shown in Figure 1A. The experimental model and timescale to investigate the adipocyte differentiation of 3T3-L1 preadipocyte is depicted in Figure 1B. At first, to examine the effects of TAK-715 on lipid accumulation during 3T3-L1 cells differentiation, different concentrations (5, 10, 15, and 20 μM) of TAK-715 were chosen and detected using Oil Red O staining. As shown in Figure 1C (upper panels), without TAK-715 (control group) in the indicated concentrations of solvent only (DMSO), no cytotoxicity to 3T3-L1 preadipocytes was noted. In addition, there was an increase in the number of stained lipid droplets (LD) in differentiated 3T3-L1 cells (D8) compared with undifferentiated cells at D0 treated with solvent only. However, the Oil Red O staining results revealed that the accumulation of LD on D8 of the differentiation in 3T3-L1 cells was inhibited following an increase in TAK-715 concentration compared with undifferentiated cells at D0. The phase-contrast view also shows the TAK-715′s inhibition effects on LD accumulation in differentiated 3T3-L1 cells (D8) (Figure 1C, lower panels). In addition, we have tested the effect of SB203580 or SB202190, another p38 MAPK inhibitors, on lipid accumulation in the differentiation of 3T3-L1 preadipocytes into adipocytes. Of interest, we have found that treatment with SB203580 or SB202190 at the designated concentrations (10 or 25 µM) strongly suppresses lipid accumulation in 3T3-L1 cells on D8 of differentiation (Supplementary Figure S1).

We next performed the AdipoRed assay to investigate whether TAK-715 reduces intracellular triglyceride (TG) levels throughout the adipogenesis of 3T3-L1 preadipocytes. As a result, TAK-715 treatment remarkably suppressed the intracellular TG levels in 3T3-L1 cells on D8 of differentiation in a dose-dependent form (Figure 1D). Eventually, the cell count analysis was performed to define whether TAK-715 at all tested concentrations has cytotoxicity towards 3T3-L1 cells. As shown in Figure 1E, TAK-715 treatment at 5 or 10 µM did not influence 3T3-L1 cells’ survival on D8 of differentiation. Nevertheless, TAK-715 at 15 and 20 μM led to a significant depletion of cell survival. Hence, the highest lipid-inhibiting effect by TAK-715 (15 or 20 μM) on D8 of 3T3-L1 preadipocyte differentiation appeared to be related to its cytotoxicity. Therefore, the 10 µM concentration of TAK-715, which revealed the most potent inhibitory effects on lipid accumulation and TG content with no cytotoxicity, was selected for subsequent studies.

### 3.2. TAK-715 Significantly Reduces the Expression and Phosphorylation Levels of C/EBP-α, PPAR-γ, and STAT-3 in 3T3-L1 Cells Following Differentiation

**Figure 2:**
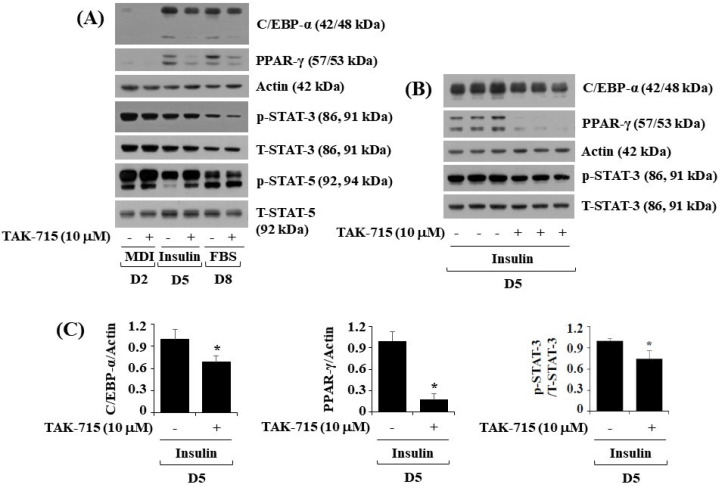
Effects of TAK-715 (10 μM) on the activation of C/EBP-α, PPAR-γ, and STAT-3/5 during 3T3-L1 cells differentiation. (**A**) 3T3-L1 preadipocyte were cultured in differentiation induction medium in the absence or presence of TAK-715 at 10 μM for days 2, 5 or 8. After the designated differentiation period, whole-cell lysates were harvested, and analyzed using western blotting analysis. (**B**) Immunoblotting analysis in triplicate experiments on day 5. (**C**) The densitometry result of (**B**) for the expression levels of C/EBP-α or PPAR-γ normalized to those of control β-actin. The bars indicate mean ± standard error (SE) of three independent experiments. * *p* < 0.05 compared to the s control samples.

To further investigate the molecular mechanisms of TAK-715-mediated reduction of lipid accumulation, 3T3-L1 preadipocytes were treated with differentiation induction medium either with or without TAK-715 (10 μM) for days 2, 5, or 8, followed by assessing transcription factors which regulate adipogenic genes, namely C/EBP-α, PPAR-γ, and STAT-3, in control or TAK-715-treated 3T3-L1 cells by using western blot analysis. On days five and eight of 3T3-L1 preadipocyte differentiation, TAK-715 treatment markedly reduced the protein expressions of C/EBP-α and PPAR-γ (Figure 2A). Additionally, TAK-715 also slightly reduced the phosphorylation levels of STAT-3 protein in 3T3-L1 cells. However, TAK-715 did not alter STAT-5 protein phosphorylation levels in 3T3-L1 cells on the days tested; instead, it increased phosphorylation levels of STAT-5 in 3T3-L1 cells differentiation, particularly on D2 and D5. Control β-actin expression and total STAT-3/5 protein levels remained stable below these experimental states. Triplicate experiments confirmed the ability of TAK-715 to significantly reduce the expression levels of C/EBP-α and PPAR-γ as well as STAT-3 phosphorylation levels in 3T3-L1 cells on day 5 of differentiation (Figure 2B). The densitometry results of Figure 2B for C/EBP-α or PPAR-γ expression levels standardized to those of control β-actin and STAT-3 phosphorylation levels normalized to those of T-STAT-3 is shown in Figure 2C.

### 3.3. TAK-715 Significantly Lower the Expressions of FAS and Perilipin A during Preadipocyte Differentiation in 3T3-L1 Cells

**Figure 3:**
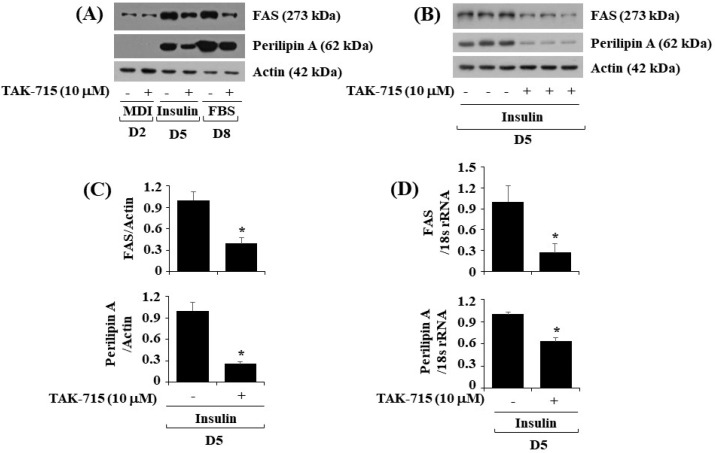
Effects of TAK-715 on the protein expression levels of FAS and perilipin A during 3T3-L1 preadipocyte differentiation. (**A**) 3T3-L1 preadipocyte were differentiated with induction medium in the absence or presence of TAK-715 (10 μM) for days 2, 5 or 8. At each point in time, whole-cell lysates were extracted, and analyzed using western blotting analysis. (**B**) Western blotting analysis in triplicate experiments on day 5. (**C**) The densitometry data of (**B**) for the protein expression levels of FAS or perilipin A normalized to those of control β-actin. (**D**) The densitometry data of real-time RT-qPCR analysis in triplicate experiments on day 5. * *p*< 0.05 compared to control on the specified day.

Next, we further explore whether TAK-715 affects the expression levels of FAS, a lipogenic enzyme, and perilipin A, an LD-binding and stabilizing protein in differentiating 3T3-L1 cells. As illustrated in Figure 3A, treatment with TAK-715 significantly down-regulated FAS and perilipin A protein expression levels in 3T3-L1 cells during differentiation days 5 and 8. Triplicate experiments results revealed the capability of TAK-715 to significantly reduce the protein expression levels of FAS and perilipin A on cell differentiation day 5 (Figure 3B). Total β-actin proteins expression level remained stable under these experimental circumstances. The densitometry results of Figure 3B are shown in Figure 3C. Real-time qPCR analysis was next carried out to see whether the reduction of FAS and perilipin A protein expression levels by TAK-715 were due to their transcriptional down-regulation during 3T3-L1 cell differentiation. In addition, triplicate experiment results exhibited that treatment with TAK-715 led to a significant reduction of FAS and perilipin A transcription on differentiation day 5 of 3T3-L1 cells.

### 3.4. TAK-715 Significantly Reduces the Phosphorylation Levels of ATF-2, a p38 MAPK Downstream Effector, in Differentiating 3T3-L1 Preadipocyte

**Figure 4:**
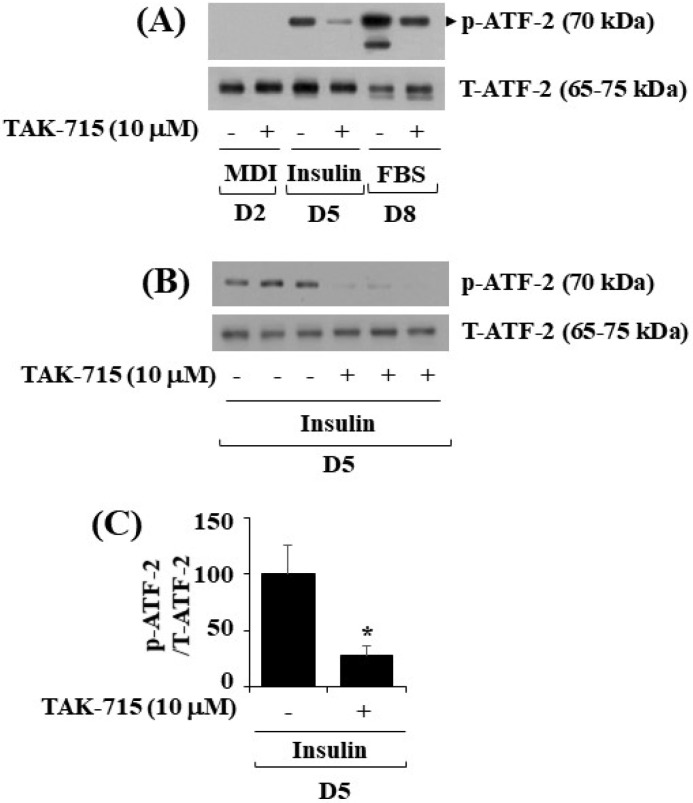
Effects of TAK-715 (10 μM) on ATF-2 phosphorylation and expression levels during 3T3-L1 preadipocyte differentiation. (**A**) For days 2, 5, and 8, 3T3-L1 preadipocytes were differentiated with induction medium in the absence or presence of TAK-715 (10 μM). At each time point, whole-cell lysates were extracted, and immunoblotting analysis was performed. (**B**) Triplicate experiments on day 5 present using immunoblotting analysis. (**C**) The densitometry data of (**B**) for ATF-2 phosphorylation levels were normalized to ATF-2 total expression levels. * *p*< 0.05 compared to the value of TAK-715 free control on the specified day.

As previously mentioned, TAK-715 is an inhibitor of p38 MAPK [12,13]. ATF-2 is a p38 MAPK downstream effector [14,15,16] and transcription factor critical in metabolic homeostasis [11,17]. We next probed whether ATF-2 is expressed and phosphorylated and whether TAK-715 regulates it during 3T3-L1 preadipocyte differentiation. Notably, the western blotting result showed that the time-dependent increase in the phosphorylation levels of ATF-2 in 3T3-L1 cells on days 5 and 8 of differentiation was blocked by TAK-715 treatment (10 µM) (Figure 4A). Experiments in triplicate further verified the capability of TAK-715 to significantly inhibit ATF-2 phosphorylation on differentiation day 5 of 3T3-L1 cells (Figure 4B). The total expression levels of total ATF-2 proteins remained stable under these experimental circumstances. The result of densitometry from Figure 4B data is depicted in Figure 4C.

Moreover, to confirm that p38 MAPK is specifically inhibited by TAK-715, we did additional experiments in which the phosphorylation (activation) level of p38 MAPK is increased by using Sorbitol, a known inducer of p38 MAPK phosphorylation [18,19]. We found herein that Sorbitol stimulated the p38 MAPK phosphorylation but TAK-715 had an ability to inhibit it in preadipocytes. These results may further ensure that TAK-715 seems to inhibit adipogenesis by blocking p38 MAPK during the preadipocyte differentiation (Supplementary Figure S2).

### 3.5. TAK-715 Markedly Reduces Lipid Formation and p38 MAPK Phosphorylation in hASCs Adipocyte Differentiation

**Figure 5:**
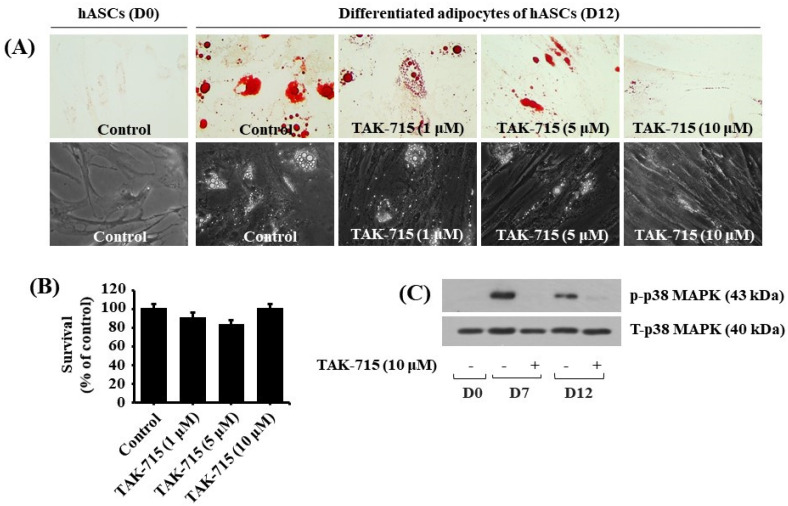
Effects of TAK-715 on lipid accumulation, cell growth (survival), and p38 MAPK phosphorylation during the adipocyte differentiation of hASCs. (**A**) Measurement of intracellular LD accumulation in undifferentiated hASCs (D0) and differentiated adipocytes (D12) that were grown with or without TAK-715 at the indicated concentrations by Oil Red O staining (upper panels) and phase-contrast microscope (lower panels) at 400× magnification. (**B**) Cell count analysis was used to visualize the amount of live cells in control or TAK-715-treated hASCs on D12 of differentiation. Results are mean ± SE from three independent experiments, each done in triplicate. (**C**) Whole-cell lysates at the desired time point were extracted and measured by western blotting analysis.

We additionally tested the effect of TAK-715 at varying concentrations (1, 5, and 10 μM) on lipid accumulation during adipocyte differentiation of hASCs. Strikingly, the Oil red O staining results demonstrated that treatment with TAK-715 led to a concentration-dependent reduction of LD accumulation in hASCs on differentiation day 12 compared with undifferentiated hASCs at D0 (Figure 5A, upper panels). TAK-715′s inhibitory effect on LD accumulation on D12 of differentiation in hASCs was also depicted using a phase-contrast microscope (Figure 5A, lower panels). Moreover, to examine whether TAK-715 at doses indicated has cytotoxicity in hASCs on D12 of differentiation using cell count assay. As shown in Figure 5B, TAK-715 treatment at concentrations tested had no cytotoxicity in hASCs on D12 of differentiation. Thus, as a result of the potent inhibitory effect on lipid accumulation with no toxicity, we selected 10 µM concentration of TAK-715 to probe further whether p38 MAPK is expressed and phosphorylated and TAK-715 regulates it during the adipocyte differentiation of hASCs. Remarkably, as illustrated in Figure 5C, there was a distinct upregulation in p38 MAPK phosphorylation levels in hASCs on days 7 and 12 of differentiation. However, TAK-715 treatment (10 μM) vastly blocked it. Total p38 MAPK expression levels remained stable under these experimental circumstances.

## 4. Discussion

The protein p38 MAPK is one of the MAPKs, and its pharmacological inhibitors have been extensively studied to demonstrate p38 MAPK’s role in obesity development. Of note, p38 MAPK regulation in adipogenesis is controversial since several studies have shown opposite results [20,21,22]. TAK-715 is a p38 MAPK inhibitor. Until now, the TAK-715′s regulation of preadipocyte differentiation and its mode of action has not been fully understood. Here, we report, for the first time, that TAK-715 at 10 µM noticeably inhibits lipid formation during adipogenesis in 3T3-L1 cells and hASCs and mediated via regulation of the expression and phosphorylation levels of C/EBP-α, PPAR-γ, STAT-3, FAS, perilipin A, ATF-2, and p38 MAPK.

It has been previously shown that SB203580 and SB202190, two different p38 MAPK inhibitors, suppress 3T3-L1 preadipocyte differentiation into adipocyte [20], supporting p38 MAPK function in adipogenesis. In agreement with this, here, we demonstrated that TAK-715 at 10 µM dramatically decreased lipid accumulation and intracellular TG form during 3T3-L1 preadipocyte differentiation without cytotoxicity. These results point out that TAK-715 has potent effects as anti-adipogenic and anti-lipogenic.

Adipogenesis is regulated by numerous genes and protein expression, which impose the phenotype of adipocyte [23]. As previously described, the transcriptional cascade and signaling pathways tightly regulate adipocyte differentiation [4]. For instance, C/EBP-δ and C/EBP-β are expressed at high levels in the early stages of preadipocyte differentiation [2]. Moreover, C/EBP-β/δ induces the expression and activation of major adipogenic transcription factors, namely C/EBP-α and PPAR-γ in the middle of adipogenesis [4]. The resultant PPAR-γ and C/EBP-α up-regulation and activation results in the differentiation and expression of adipocyte-specific genes, such as aP2, LPL, FAS, and perilipin A, during the late stage of preadipocyte differentiation [2]. It is also worth stating that STAT-3 is essential in the initial stages of adipogenesis [24,25,26]. Given the current findings that TAK-715 significantly reduces the expression levels of C/EBP-α and PPAR-γ as well as STAT-3 phosphorylation levels during 3T3-L1 preadipocyte differentiation, TAK-715′s lipid-lowering effect on 3T3-L1 cells is most likely due to the reduction of C/EBP-α, PPAR-γ, and STAT-3 activation. A wealth of information indicates that lipid accumulation or storage during the differentiation of a preadipocyte into an adipocyte is also marked by the upregulation of enzymes that have a role in lipogenesis, such as FAS and lipid droplet-binding and stabilizing proteins like perilipin A [27,28,29]. FAS is an enzyme highly expressed in various tissues, including adipose tissue, to catalyze fatty acid synthesis [27]. Perilipin A is found exclusively on the LD outer surface and acts as a protective barrier to the LD once the preadipocyte becomes differentiated into mature adipocytes without stimulation [28]. Of interest, the present study illustrated that TAK-715 can greatly down-regulate the expression levels of mRNA as well as FAS and perilipin A in differentiating 3T3-L1 cells. Thus, the TAK-715′s strong lipid-lowering (anti-lipogenic) effect is further linked to FAS and perilipin A expression levels inhibition during 3T3-L1 preadipocyte differentiation.

ATF-2 is a family member of the ATF transcription factor that regulates metabolic homeostasis [11,17]. Reportedly, stress-activated protein kinases (SAPKs), namely p38 MAPK, phosphorylate and activate ATF-2 at Thr 69 and Thr 71 [11]. It is of interest that the results demonstrated that ATF-2 plays a positive role in adipogenesis by activating the transcription of the phosphoenolpyruvate carboxykinase-cytosolic (PEPCK-C) via the p38 MAPK pathway [9], and it promotes the initial adipogenesis by regulating PPAR-γ and C/EBP-β expression [30,31,32]. TAK-715 was shown in this study to essentially block ATF-2 phosphorylation in differentiating 3T3-L1 cells, supporting the drug’s efficacy in inhibiting the p38 MAPK/ATF-2 pathway. Further, considering that ATF-2 is a p38 MAPK downstream effector and acts as a transcription factor controlling PPAR-γ and C/EBP-β expression, it is also speculative that TAK-715 may exert its anti-adipogenic effects via the p38 MAPK/ATF-2-dependent regulation of PPAR-γ and C/EBP-β during 3T3-L1 preadipocyte differentiation into adipocyte. It will be interesting to examine whether shRNA-based gene silencing of p38 MAPK and/or ATF-2 influences the expression of PPAR-γ and/or C/EBP-β (and other adipogenic markers) during the 3T3-L1 preadipocyte differentiation process in the future.

The current study also discovered that TAK-715 regulates the adipocyte differentiation of human adipose stem cells (hASCs) via the suppression of p38 MAPK. Little is known about the phosphorylation and expression and function of p38 MAPK in hASCs and their differentiation process. We herein demonstrated that p38 MAPK is substantially expressed and highly but transiently phosphorylated during the adipocyte differentiation of hASCs. Moreover, the present study illustrated that TAK-715 at 10 μM, which strongly blocks p38 MAPK phosphorylation, also markedly suppresses lipid accumulation during the adipocyte differentiation of hASCs, which may thus support a positive role of p38 MAPK in lipid accumulation in the hASCs adipocyte differentiation. Notably, there were previous studies displaying that WNT/β catenin signaling activates p38 MAPK, which plays a pivotal role for triggering the adipocyte differentiation of mesenchymal stem cells [33,34]. These results may further imply the significance of the WNT/β catenin-p38 MAPK signaling axis during the adipocyte differentiation of hASCs. It will be interesting, in the future, to investigate the expression and role of WNT/β catenin during the adipocyte differentiation of hASCs through the regulation of p38 MAPK.

There were multiple studies that show that the increase of p38 MAPK indirectly relates to the alteration of the lipid metabolism through the enzyme acyl-CoA (cholesterol acyl transferase (ACAT) activity) [35]. ACAT plays a role in cholesterol homeostasis by storing it in the form of lipid droplet [36]. In normal adipose tissue, ACAT expression and activity remains very low. However, the overexpression of ACAT were demonstrated in obese mice which leads to adipose tissue dysfunction [37]. Hence, the decrease of intracellular TG content and LDs in TAK-715-treated 3T3-L1 cells and hASCs during their differentiation process seems to be associated with the ACAT activity, which needs to be further investigated in the future research.

**Figure 6:**
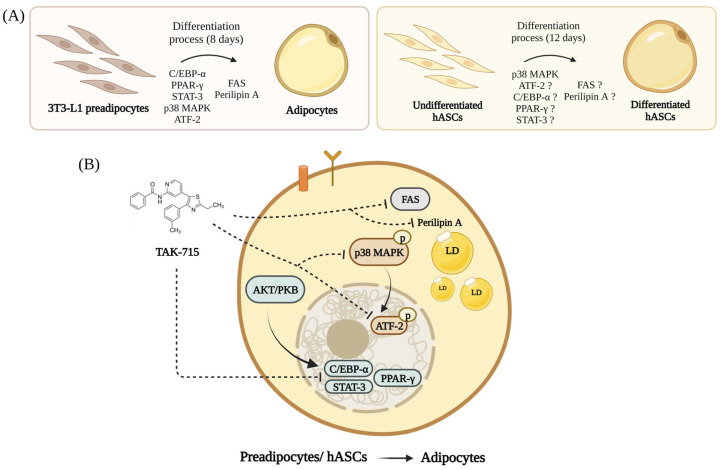
A graphical abstract showing the TAK-715′s anti-adipogenic effect and mechanism during the adipocyte differentiation of 3T3-L1 cells and hASCs (Created with BioRender.com). (**A**) Adipogenic genes expression are progressively increases in differentiating 3T3-L1 preadipocytes and hASCs. (**B**) The molecular and cellular mechanisms underlying the TAK-715′s anti-adipogenic effect on 3T3-L1 cells and hASCs.

Based on our findings here, we propose a graphical model for TAK-715 regulation in adipogenesis of 3T3-L1 preadipocytes and hASCs. As shown in Figure 6A, preadipocytes can be induced to differentiate into mature adipocytes. Our results demonstrated in Figure 6B that (1) TAK-715 reduces the expression of adipogenesis-related transcription factors such as C/EBP-α, PPAR-γ, and STAT-3/5 in differentiating 3T3-L1 cells and hASCs; (2) TAK-715 decreases the activation (phosphorylation) of p38 MAPK during the adipocyte differentiation of 3T3-L1 cells and hASCs; (3) TAK-715 decreases the phosphorylation of ATF-2 in differentiating 3T3-L1 cells; (4) TAK-715 inhibits the expression of FAS, a lipogenesis-related marker, during the preadipocyte differentiation; and (5) TAK-715 down-regulates the expression of perilipin A, a LDs stabilizing protein, in differentiating 3T3-L1 cells.

To summarize, this is the first report to demonstrate that TAK-715 has potent lipid-lowering effects during the adipocyte differentiation of 3T3-L1 preadipocytes and hASCs, which are mediated by the regulation of C/EBP-α, PPAR-γ, STAT-3, FAS, perilipin A, p38 MAPK, and ATF-2.

## 5. Conclusions

The current study illustrates the ability of TAK-715 to strongly inhibit lipid accumulation during the differentiation of 3T3-L1 preadipocytes and hASCs into adipocytes, which may thus point out the drug’s application as a potential preventive or therapeutic agent for treating obesity and its associated disorders.

## Figures and Tables

**Table 1:** List of antibody details used in western blot analysis.

Antibodies	Dilution Used	Source	Catalog No.
*Primary Antibodies*			
C/EBP-α	1:2000	Santa Cruz Biotechnology	sc-61
PPAR-γ	1:2000	Santa Cruz Biotechnology	sc-7272
p-STAT-3	1:2000	Santa Cruz Biotechnology	sc-8059
T-STAT-3	1:2000	Santa Cruz Biotechnology	sc-8019
p-STAT-5	1:2000	Santa Cruz Biotechnology	sc-10180b
T-STAT-5	1:2000	Santa Cruz Biotechnology	sc-835
FAS	1:2000	BD Bioscience	#9452
Perilipin A	1:2000	BioVision	#3948-200
p-ATF-2	1:2000	Cell Signaling	#9225
T-ATF-2	1:2000	Cell Signaling	#9226
p-p38 MAPK	1:2000	Cell Signaling	#9211
T-p38 MAPK	1:2000	Cell Signaling	#9212
β-actin	1:10,000	Sigma	A5441

**Table 2:** Sequences of primers used for quantitative real-time PCR.

Gene	Forward	Reverse
FAS	TTGCTGGCACTACAGAATGC	AACAGCCTCAGAGCGACAAT
Perilipin A	CTTTCTCGACACACCATGGAAAC	CCACGTTATCCGTAACACCCTTCA
18S rRNA	GGTGAAGGTCGGTGTGAACG	GGTAGGAACACGGAAGGCCA

## Data Availability

Data is contained within the article. All data are available upon request from the corresponding author.

## Supplementary Materials

The following supporting information can be downloaded at: https://www.mdpi.com/article/10.3390/life13020412/s1, Figure S1: SB203580 and SB202190 effect on lipid droplet accumulation during the adipogenesis of 3T3-L1 cells; Figure S2: Effects of TAK-715 on Sorbitol-induced p38 MAPK phosphorylation in 3T3-L1 preadipocytes.
