# Supplementary material for: Suppression of Lipid Accumulation in the Differentiation of 3T3-L1 Preadipocytes and Human Adipose Stem Cells into Adipocytes by TAK-715, a Specific Inhibitor of p38 MAPK
Source: Life (Basel). 2023 Feb 1;13(2):412. doi: 10.3390/life13020412 (PMC9965126; doi:10.3390/life13020412)

Supplementary Information

Supplementary Figure S1: SB203580 and SB202190 effect on lipid droplet accumulation during the adipogenesis of 3T3-L1 cells. Representative images of intracellular lipid droplet (LD) formation in 3T3-L1 preadipocyte (D0) and adipocyte (D8) that were grown in the absence or presence of SB203580 (10 and 25 μM) or SB202190 (25 μM) by phase-contrast image at 400 x magnification.

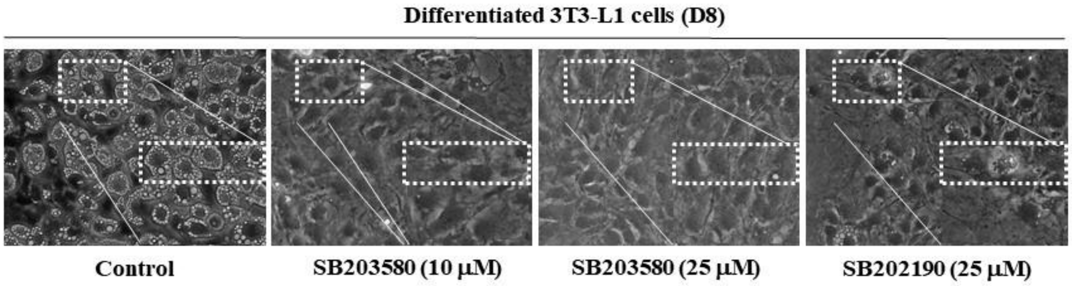

Supplementary Figure S2: Effects of TAK-715 on Sorbitol-induced p38 MAPK phosphorylation in 3T3-L1 preadipocytes. 3T3-L1 preadipocytes were treated without or with Sorbitol (0.5 mM) in the presence or absence of TAK-715 at 10 μM for 0.5 h. Whole-cell lysate was prepared and analysed by Western blotting. p-p38 MAPK, phosphorylated p38 MAPK; T-p38 MAPK, total p38 MAPK.

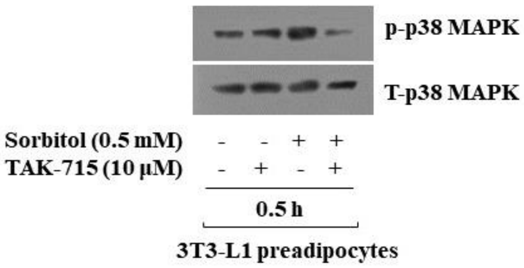

Supplement: Supplementary file 1 [file life-13-00412-s001.zip › life-2157474-supplementary.pdf]
